# Comparison of clinicopathological parameters with the presence of Epstein–Barr virus and the absence of DNA mismatch repair proteins in gastric adenocarcinomas

**DOI:** 10.2478/abm-2025-0011

**Published:** 2025-04-30

**Authors:** Özge Eyeoğlu, Serra Kayaçetin

**Affiliations:** 1 Department of Clinical Pathology, Gaziantep City Hospital, Gaziantep 27000, Turkey; 2 Department of Clinical Pathology, Bilkent City Hospital, Ankara 06800, Turkey

**Keywords:** Epstein–Barr virus, microsatellite instability, prognosis, stomach cancer, tumor microenvironment

## Abstract

**Background:**

High mortality and poor prognosis are seen in gastric adenocarcinomas (GAs). Therefore, investigation of the factors related to GA prognosis is important.

**Objective:**

To investigate the association between clinicopathological parameters and DNA mismatch repair (MMR) proteins as well as Epstein–Barr virus (EBV) in GAs.

**Methods:**

Expression of MMR proteins and EBV positivity of 77 patients diagnosed with GA were evaluated using immunohistochemistry. Survival data of the patients were also considered.

**Results:**

Significant correlations were found between EBV positivity and gender, perineural invasion (PNI), and histological type. PNI was less common in EBV-positive patients, and EBV positivity was highly correlated with lymphoid stromal adenocarcinoma. Tumor budding was significantly correlated with histological type and grade, lymphovascular invasion (LVI), PNI, lymph node metastasis, and post-diagnosis survival time. Moreover, tumor–stroma ratio was correlated with tumor stage. Additionally, tumor location, histological grade, tumor budding, PNI, and pathological stage were associated with survival. Also, EBV positivity was significantly associated with histological type, PNI, tumor location, and gender. However, MMR and EBV positivity were not significantly correlated to tumor microenvironment and prognosis. It was noteworthy that the mortality rate was much higher in patients with PNI compared with those without PNI.

**Conclusion:**

Our findings support that the tumor microenvironment is significantly associated with GA prognosis.

Gastric adenocarcinoma is the 5th most frequently diagnosed tumor in the world and the 3rd most frequently diagnosed cancer-related death in the world [1]. The incidence of gastric cancer is the highest in the Far East, Central Asia, and Latin America, while it is the lowest in the northern and eastern regions of Africa. The risk of developing gastric cancer is 1.87% in men and 0.79% in women. It is 2.2 times more common in men than in women in the developed countries and 1.83 times more common in developing countries [2].

Environmental and genetic factors play an important role in the pathogenesis of gastric adenocarcinoma. Most patients are >50 years of age. Malignancy may also develop in young patients after chemotherapy and irritation [3]. In practice, chronic atrophic gastritis with intestinal metaplasia and dysplasia is the basis of most gastric cancers. In hypocaloric patients, high pH causes bacterial growth and may lead to the development of gastric carcinoma over the years. Again, gastric carcinoma may develop in patients with pernicious anemia. The most important etiological factor identified is *Helicobacter pylori*. It causes the development of intestinal-type gastric carcinoma, the most common subtype according to the Lauren classification [4]. The Cancer Genome Atlas has classified gastric cancers into 4 molecular subgroups: Epstein–Barr virus (EBV)-positive, microsatellite instable, genomically instable, and chromosomally stable [5]. Menetrier disease, gastric peptic ulcer, and gastric stumps are important factors in the pathogenesis of gastric cancer. According to recent studies, 6%–7% of gastric cancers in Japan and 16% in England have been associated with EBV [6]. Most EBV-positive gastric cancers are histologically lymphoid stroma-rich or lymphoepithelioma-like. Mutations in phosphatidylinositol-4, 5-bisphosphate 3-kinase catalytic subunit alpha (*PIK3CA*) and AT-rich interaction domain 1A (*ARID1A*) show genome-wide hypermethylation and amplification of the programmed cell death ligand 1 (*PD-L1*) gene, an important immune check-point regulator [7]. Mutations in DNA repair genes, promoter region methylation, or DNA-wide hypermethylation are seen in microsatellite instable gastric cancers [8].

Mismatch repair (MMR) includes a number of DNA mismatch repair enzymes: MutL homolog 1 (MLH1), MutL homolog 3 (MLH3), MutS homolog 2 (MSH2), MutS homolog 3 (MSH3), MutS homolog 6 (MSH6), post meiotic segregation 1 (PMS1), and post meiotic segregation 2 (PMS2). During normal DNA replication, the heterodimeric complexes, MSH2/MSH6 and MSH2/MSH3, detect and bind small DNA mismatch errors, while the MLH1/PMS2 heterodimers are responsible for excision and resynthesis of corrected DNA bases at the mismatch sites. Loss of expression of one or more DNA mismatch repair enzymes results in the deficiency of the complex and consequent failure of DNA repair. Microsatellites are repeats of 1–6 nucleotides spread throughout the genome, prone to mutation. In the presence of MMR deficiency, an extremely variable phenotype is observed, and the resulting microsatellite instability (MSI) is associated with gastric cancer [9].

In this study, we investigated the relationship between MMR/MSI and EBV positivity, histological and clinical parameters including histological type, histological grade, patient age, gender, TNM (tumor, extent of spread to the lymph nodes, and presence of metastasis) class, survival time after diagnosis, lymphovascular invasion (LVI), and perineural invasion (PNI), as well as the tumor microenvironment in gastric cancer patients with different stages and histology. The aim of this study was to determine the importance of current parameters in terms of future targeted therapies in gastric cancers with high mortality and poor prognosis and to reveal the relationship of these parameters with survival.

## Methods

The study protocol was reviewed and approved by the Institutional Review Board of Ankara Bilkent City Hospital (date: July 19, 2023 and number: E2-23-4274), and was conducted in accordance with the 1964 Helsinki Declaration and its amendments or comparable ethical standards. All the study participants provided informed consent.

Blocks and slides of 87 patients who were diagnosed with primary advanced gastric adenocarcinoma after total and subtotal gastrectomy operations in Ankara Bilkent City Hospital (Turkey) between January 2021 and December 2022 were examined. Of these, 10 cases were not included in the study because the tumor area was too small. Selected tumor samples from 77 patients were taken from tissues that were as free of necrosis as possible, contained live tumor areas, and had good paraffin follow-up. None of the patients received neoadjuvant treatment. Clinical and some pathological data of the patients (age, gender, tumor location, TNM class, histological type and grade of the tumor, LVI, PNI, lymph node metastasis and stage) were obtained from the pathology reports of the patients in our hospital database.

Hematoxylin and eosin (H&E) and immunohistochemistry stained slide blocks, if any, belonging to the patients in the department archive were extracted from our hospital pathology archive. The samples were blindly evaluated again in terms of histological subtype, histological grade, PNI, LVI, tumor budding, metastatic lymph node count, total lymph node count, and pathological tumor stage by two observers under the microscope. In addition, the tumor microenvironment evaluation, which was not previously reported, was examined in terms of tumor budding, tumor–stroma ratio (TSR), and the presence of peritumoral lymphocytes infiltrating the tumor.

### Tissue microarray

For each sample, the areas with good fixation, sufficient tumor tissue, and little necrosis were marked on the slide and block. The marked area was scraped from the block with a 5-mm punch biopsy tool and mapped onto previously prepared empty paraffin blocks in our laboratory, and embedded in each block with 7 samples. A total of 11 paraffin blocks were prepared and tissue microarray (TMA) was performed. The prepared blocks were kept in a 40°C oven for 24 h. Then, unstained sections of 4 μm thickness were taken from the blocks for immunohistochemical studies.

### Immunohistochemistry

The paraffin-embedded tissues were deparaffinized using a Bond-Max device (Leica). MSH2 (RBT-MSH2, RMab, Bio SB), MSH6 (44, MMab, Bio SB), MLH1 (RBT-MLH1, RMab, Bio SB), PMS2 (RBT-PMS2 RMab, Bio SB), and EBV LMP (Agilent Technologies) primary antibodies were used for immunohistochemistry. Retrieval solution was boiled in ethylenediaminetetraacetic acid (EDTA) for 20 min and kept in primary antibody for 30 min. Horseradish peroxidase (HRP)-conjugated Bond Polymer Refine Detection Kit (Leica) was used for the detection of primary antibodies according to the manufacturer’s recommendations.

### Statistical analysis

Statistical analyses were conducted using SPSS Statistics for Windows version 17 program (IBM Corp.). In all statistical analyses, the test method was selected considering the type of parameters. The Spearman rank correlation method and Kruskal–Wallis were used to search for a relationship. If both parameters contained ordinal data, the Kendall rank correlation method was used. If the data in both parameters were binary, the Fisher transformation method was used. The Log-rank test was used for survival association. Curves were created using the Kaplan–Meier method to test the result obtained. For all remaining cases, the *P*-value was calculated using Cramer’s V (Φc) statistic. In statistical analyses, the significance level of the tests was decided by comparing them with the value of *P* < 0.05.

## Results

### Demographic and clinicopathological characteristics

The mean age of patients was 62.4 ± 11.5 (min: 19, max: 88) years, and 63.64% of the patients (n = 49) were male while 36.36% (n = 28) were female. The mean survival time was 17.1 ± 10.0 (min: 0, max: 36) months. Clinical and pathological characteristics of the patients are given in Table 1. The highest number of locations was found to be the corpus of the stomach with a rate of 55.84% (n = 43), histological type was classical (28.57%, n = 22), histological grade was moderate (49.35%, n = 38), tumor budding was grade 1 (37.66%, n = 29), LVI was present in 60 patients (77.92%), PNI was observed in 48 patients (62.34%), lymph node metastasis was seen in 52 patients (67.53%), tumor stage was 3A in 23 patients (29.87%), MSI/MMR was preserved in 59 patients (76.62%), 68 patients (88.31%) were EBV-negative, TSR was high in 60 patients (77.92%), and the presence of tumor-infiltrating and peritumoral lymphocytes was grade 3 in 44 patients (57.14%).

**Table 1.** Clinical and pathological characteristics of the patients included in this study

Variables		Number	Percent
Gender	Male	49	63.64
	Female	28	36.36
Tumor location	Corpus	43	55.84
	Cardia	14	18.18
	Antrum	9	11.69
	Multiple	6	7.79
	Pylorus	5	6.49
Histological type	Classic	22	28.57
	Less cohesive	21	27.27
	Mixed	20	25.97
	Papillary	6	7.79
	Lymphoid stroma	4	5.19
	Tubular	3	3.90
	Mucinous	1	1.30
Histological grade	Moderately	38	49.35
	differentiated		
	Poorly differentiated	33	42.86
	Well differentiated	6	7.79
Tumor budding	Grade 1	29	37.66
	Grade 2	17	22.08
	Grade 3	31	40.26
LVI	Present	60	77.92
	Absent	17	22.08
PNI	Present	48	62.34
	Absent	29	37.66
Lymph mode metastasis	Present	52	67.53
	Absent	25	32.47
Stage	1A	7	9.09
	1B	2	2.60
	2A	13	16.88
	2B	7	9.09
	3A	23	29.87
	3B	8	10.39
	3C	13	16.88
	4A	4	5.19
MMR/MSI	Preserved	59	76.62
	Lost	18	23.38
EBV	Negative	68	88.31
	Positive	9	11.69
TSR	Low	17	22.08
	High	60	77.92
Tumor infiltrating and peritumoral lymphocyte presence	Grade 1	16	20.78
	Grade 2	17	22.08
	Grade 3	44	57.14

1 EBV, Epstein–Barr virus; LVI, lymphovascular invasion; MMR, mismatch repair; MSI, microsatellite instability; PNI, perineural invasion; TSR, tumor–stroma ratio.

### Relationship of EBV with other parameters

Out of 77 patients, 9 patients (11.69%) were EBV-positive while 68 patients (88.31%) were EBV-negative.

A strong relationship was found between the EBV and histological type parameters (Φc = 0.67). The “less cohesive,” “classic,” “mixed,” and “tubular” histological types showed a similar distribution and were mostly EBV-negative. All “lymphoid stroma” types were EBV-positive, while all “mucinous” and “papillary” types were EBV-negative.

A moderate level of relationship was found between EBV and gender (Φc = 0.27). EBV is often negative in male patients, while a more balanced distribution was found in female cases. Similarly, the relationship was at a moderate level between the EBV and PNI parameters (Φc = 0.26). A high rate of EBV negativity was detected in cases with PNI. However, no significant relationship was found between EBV and location, histological grade, tumor budding, LVI, lymph node metastasis, TSR, pathological stage, and presence of tumor infiltrating peritumoral lymphocytes.

### Relationship of tumor budding with other parameters

The tumor budding parameter was examined as grades 1, 2, and 3. The highest was grade 3 with 31 (40.0%) cases. There were grade 1 in 29 (38.0%) and grade 2 in 17 (22.0%) patients.

A significant relationship was found between the parameters of tumor budding and histological grade (*P* = 0.00132). Most of the tumors with grade 1 tumor budding are tumors with a “moderately differentiated” histological grade. In cases with grade 3 budding, there is mostly a “poorly differentiated” histological grade. No “well differentiated” histological grade is seen for grade 3. If tumor budding increases from grade 1 to grade 3, the histological grade also tends from “well differentiated” to “poorly differentiated.” A significant relationship was also found between the tumor budding and PNI (Φc = 0.54). While PNI was not observed in most cases with tumor budding grade 1, PNI was observed in most cases with grade 3. Additionally, there was a significant relationship between tumor budding and stage (*P* = 0.01632). Grade 1 was mostly observed with stages 1A and 2A. In patients with stage 3C, grade 3 was predominant. Kendall’s Tau value for these two parameters was calculated as 0.34, which shows that if stage values go from 1A to 3C, the value in tumor budding also tends to go from grade 1 to grade 3.

Tumor budding and histological type parameters were found to be moderately related (Φc = 0.44). Grade 3 was mostly seen in tumors with the histological type “less cohesive.” A similar relationship was found between grade 1 budding and “classic” histological type. On the contrary, only grade 1 budding was observed in “mucinous,” “papillary,” and “tubular” histological types. Similarly, tumor budding and LVI parameters were found to be related moderately (Φc = 0.42). In grades 2 and 3 budding, the presence of LVI was observed more dominantly than in grade 1 budding. However, tumor budding was weakly related (Φc = 0.37) with lymph node metastasis. When lymph node metastasis was present, grades 3 and 2 tumor budding were more common. In grade 1 tumor, budding and lymph node metastasis values were equally distributed.

### Relationship of TSR with other parameters

TSR was examined in two groups as “high” and “low,” which was seen in 60 (68.0%) and 17 (22.0%) patients, respectively. A statistically significant (*P* = 0.034) relationship was only found between the TSR and tumor stage at a moderate level (Φc = 0.44). In stage 3A and 3C patients, the TSR was seen as “high.”

### Relationship of MSI/MMR with other parameters

MSI/MMR status was examined in two groups as lost and preserved. MSI/MMR was preserved in 59 (77.0%) and lost in 18 (23.0%) patients. As a result of the analyses, the relationship between MSI/MMR and other parameters was not statistically significant.

### Relationship of tumor-infiltrating and peritumoral lymphocytes with other parameters

The presence of tumor-infiltrating and peritumoral lymphocytes was examined as grades 1, 2, and 3. Of the 77 patients, 16 (21.0%) were observed as grade 1, 17 (22.0%) as grade 2, and 44 (57.0%) as grade 3. The results showed that a statistically significant relationship was not found between the presence of tumor-infiltrating and peritumoral lymphocytes and other parameters.

### Relationship of survival time with other parameters

**Figure 1.**
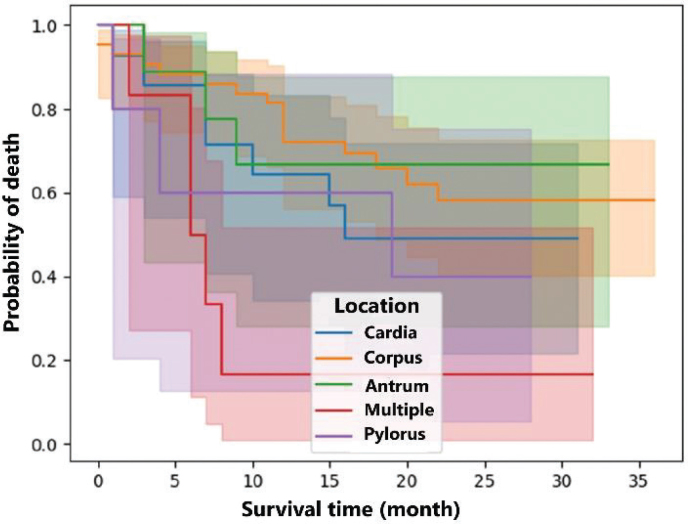
The Kaplan–Meier survival curve showing the relationship between tumor location and survival time.

The relationship between location and survival time is provided in Figure 1. The highest survival time was observed in patients with antrum location, while the lowest survival time was observed in patients with multiple tumors. In 9 patients with antrum location, the average survival time was 20.22 months, which is quite high compared with other locations. The median for antrum was observed as 22 months. In the case of multiple locations, the average survival time was calculated as 10.17 months.

**Figure 2.**
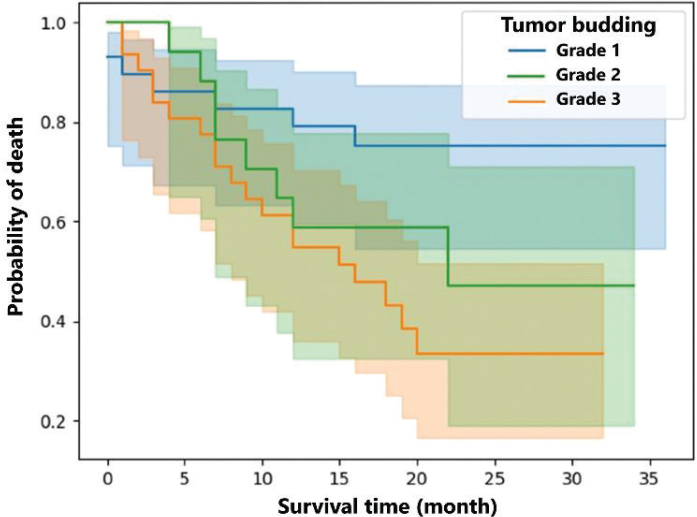
The Kaplan–Meier survival curve showing the relationship between tumor budding and survival time.

The relationship between tumor budding and survival time is seen in Figure 2. The budding type with the highest survival time is grade 1. While the average survival time for tumor budding grade 1 was 21.14 months, it was 16.24 months for grade 2 and 13.87 months for grade 3. Additionally, in the patient group with no PNI, a higher survival time was observed compared with the patient group with PNI. Moreover, the stages with the lowest survival time were 3B and 3C. For stage 3B, the mean survival time of 8 patients was 12.3 months and the median value was 9.5 months. In stage 3C including 13 patients, the mean and median values were around 13 months.

## Discussion

Gastric adenocarcinomas (GAs) are tumors with high mortality and poor prognosis [10]. The importance of molecular pathways is increasing in the diagnosis and treatment [3]. In the literature, gastric carcinoma was found to be more common in male patients than in females and the mean age of the cases is over 60 [11]. In this study, the mean age was found to be 62.4 years, consistent with the literature, and was more common in male patients (63.64%) than in females (36.36%).

The most effective genetic pathways facilitating checkpoint immunotherapy are thought to be immune activity-related ARID1A and PIK3CA mutations [12]. Phosphatidylinositol 3’ -kinase (PI3K) pathway activity was also found to be associated with antitumor immunity in gastric cancer patients [13, 14]. In addition, some studies have shown that regulation of immune-related signaling, increased density of tumorinfiltrating immune cells, and enhanced immune recognition of tumor cells are correlated with pharmacological inhibition of the PI3K pathway [15]. It is very important to distinguish patients who may benefit from immunotherapy from others in terms of treatment approach. Biomarkers that have been proposed to predict potential response to immune checkpoint inhibitors include MMR and/or EBV status [11, 16]. According to this study, the frequency of MMR/MSI (23.38%) and EBV (11.69%) in gastric cancers is consistent with the latest The Cancer Genome Atlas Program (TCGA) data [5].

Although the mechanisms of how EBV infection leads to EBV-associated gastric cancer are still unclear, it is proposed that EBV-positive gastric cancers cause some gene amplifications, leading to carcinogenesis through various molecular pathways, especially PIK3CA [17]. In addition, EBV positivity leads to a high tumor burden stimulating host immune cells in large amounts, which is associated with an increase in immune cells in the peritumoral or intratumoral tissue [12]. Therefore, EBV-positive gastric cancer patients may benefit from immune checkpoint inhibitors [18].

Gastric cancer with lymphoid stroma (GCLS) constitutes 1%–4% of all gastric cancers and most cases are EBV-positive. The relationship between EBV positivity in gastric cancers and the GCLS subtype was first described by Burke et al. [19]. GCLS is most likely associated with latent EBV infection. However, EBV can also be positive in gastric cancers with conventional histology. Therefore, EBV should be studied in all gastric cancer cases to detect all EBV-positive gastric cancers. Moreover, EBV-negative GCLS was suggested to have a more aggressive course compared with conventional gastric cancers [20]. It would be interesting to characterize tumor-infiltrating lymphoid cells and their microenvironment in both EBV-positive and EBV-negative GCLS and to further investigate the underlying mechanisms.

## Conclusions

In this study, in accordance with the literature, the lymphoid stroma subtype and tumor location was found to be significantly associated with EBV positivity. However, in contrast to the literature, we found that the EBV prevalence was 3.5 times higher in female patients than in male patients. This might be due to the unequal female–male ratio in our patient group and patient heterogeneity. Interestingly, we also showed that PNI was significantly more common in EBV-negative patients. In previous studies, no significant difference was found between EBV-positive and EBV-negative patients in terms of the presence/absence of PNI [21, 22].

In addition to EBV and MMR/MSI biomarkers, evaluation of the tumor microenvironment in gastric cancer patients has also been shown to be an important indicator of response to immune checkpoint therapy [12, 17]. In this study, tumor budding was found to be associated with histological subtype, histological grade of tumor, LVI, PNI, lymph node metastasis, and pathological stage, in accordance with the existing literature [17]. In addition, we found that TSR was associated with pathological stage, and high TSR was detected in patients with high pathological stage, as reported previously [12, 17].

There are some limitations to this study. First, this study was single-centered and we were able to include only 77 cases in this study due to the limited number of gastric cancer patients admitted to our hospital, the limited number of blocks that could be accessed from the archive, and the inadequacy of tumor tissue in some gastric cancer cases. Another limitation of this study is that we could not evaluate the relationship between EBV and MMR/MSI status and PD-L1 in gastric cancer patients due to various reasons. It would be more interesting to evaluate these data in subsequent studies.

One of the most important points of this study is that MMR/MSI and EBV status were calculated separately using different statistical methods with the tumor microenvironment and prognostic data. Studies that are similar to our analysis are available [23]. However, larger studies are needed to clarify the relationship between MMR/MSI and EBV status, prognostic factors, and tumor microenvironment. The results may contribute to the understanding of genetic and epigenetic processes of tumorigenesis in MMR/MSI and/or EBV-positive gastric cancers.
